# Multivariable Associations of Anthropometric and Psychosocial Markers with an Elevated Abdominal Volume Index Among University Students: A Sex- and Academic Program-Stratified Analysis

**DOI:** 10.3390/healthcare14152395

**Published:** 2026-08-04

**Authors:** Ioana Mihaela Tomulescu, Mihai Ion Marian, Ciprian Brisc

**Affiliations:** 1 Department of Biology, Faculty of Informatics and Sciences, University of Oradea, 410087 Oradea, Romania; 2 Doctoral School of Biomedical Sciences, University of Oradea, 410087 Oradea, Romania; briscipian@gmail.com; 3 Department of Psychology, Faculty of Social Sciences and Humanities, University of Oradea, 410087 Oradea, Romania; 4 Bihor Clinical County Emergency Hospital, 410169 Oradea, Romania; 5 Department of Medical Disciplines, Faculty of Medicine and Pharmacy, University of Oradea, 410071 Oradea, Romania

**Keywords:** abdominal adiposity volume, anthropometric indices, anthropometric covariates, 2D:4D digit ratio, perceived stress, non-invasive screening, university students

## Abstract

**Highlights:**

**What are the main findings?**

- Traditional anthropometric parameters demonstrate classification alignment with the Abdominal Volume Index (AVI), serving as baseline covariates within the studied cohort of young adults.
- The multi-variable association profiles reveal distinct sex-based variations, with biological and psychological parameters maintaining statistical significance (*p* ≤ 0.001) primarily within the analyzed female student sub-cohort.

**What are the implications of the main findings?**

- Non-invasive risk-assessment protocols tailored for university health settings may benefit from incorporating prenatal markers (2D:4D ratio) and academic stressors alongside standard physical measurements.
- Screening frameworks for metabolic vulnerabilities within this demographic suggest the utility of simultaneous anthropometric and psychological evaluations to account for sex-specific co-variation.

**Abstract:**

**Background:** The Adiposity Volume Index (AVI) is a modern geometric tool for tracking central body fat. However, how it interacts with prenatal markers (2D:4D ratio), advanced anthropometric indexes, and psychological factors across different academic disciplines remains largely unexplored. This study evaluates the sex-specific association profiles and screening capacity of these parameters in healthy university students. **Methods:** A cross-sectional study was conducted on 445 students (199 men and 246 women) from Biomedical Sciences (BS), Computer Science and Engineering (CSE), and Social Sciences and Physiotherapy (SSP) academic programs. Anthropometric indicators (Body Mass Index—BMI, Body Surface Area—BSA, Weight-adjusted Waist Index—WWI, Sagittal Abdominal Diameter-to-Waist circumference Ratio—SAD-to-Waist Ratio), the right-hand digital 2D:4D ratio (R-2D:4D), and Perceived Stress Questionnaire scores (PSQ) were assessed. Data were analyzed using multi-layered linear regression and ROC curve analysis. **Results**: In male students, BMI, BSA and WWI consistently co-varied with AVI across all academic programs (*R*^2^ > 0.978, *p* < 0.001). In contrast, a major psychosomatic interaction emerged in female students. Among stressed BS students, the linear association model shifted (*R*^2^ = 0.313, lost significance), and the prenatal R-2D:4D ratio became a significant negative independent correlate (*B* = −87.780, *p* = 0.019). ROC analysis revealed that optimal BMI cut-offs for elevated AVI were 26.96 kg/m^2^ (*AUC* = 0.957) for men and 24.89 kg/m^2^ (*AUC* = 0.972) for women. Notably, perceived stress was a significant diagnostic marker only for female students (*AUC* = 0.621, *p* < 0.001, *cut-off* = 65.50). **Conclusions:** Perceived academic stress exhibits a significant concurrent association with abdominal fat distribution patterns exclusively in female biomedical students, co-varying with prenatal endocrine markers within the studied cohort. Furthermore, AVI-driven screening demonstrates that metabolic vulnerabilities in these young adults may accumulate within the conventional normal-weight BMI range for women, emphasizing the potential utility of sex-tailored clinical assessments.

## 1. Introduction

Overweight and obesity are the most prevalent metabolic disorders in developed countries. The prevalence of obesity has increased tremendously in recent decades [1]. Obesity is a major risk factor for the development of multimorbidity, and the prevalence of obesity continues to rise worldwide [2]. Obesity refers to an excessive or abnormal accumulation of body fat, which adversely affects health [3]. Obesity ranks as the sixth most significant risk factor contributing to the global burden of illness [4]. Abdominal obesity (central or visceral) is an important risk factor for cardiovascular diseases, diabetes, and cancer, playing a critical role in the so-called metabolic syndrome [1]. The obese phenotype is highly complex, and in certain cases, patients do not display obvious cardiometabolic symptoms. Consequently, in these instances, obesity—particularly abdominal obesity, whether central or visceral—induces or even exacerbates insulin resistance. This insulin resistance, in turn, triggers various metabolic imbalances that culminate in the development of the clinical entity known as metabolic syndrome [5].

Obesity is typically diagnosed based on the Body Mass Index (BMI), and the incidence of numerous chronic diseases, such as hypertension, diabetes, dyslipidemia, and metabolic syndrome, increases substantially with a higher BMI [6,7]. However, the simple anthropometric indicator BMI cannot provide nuanced insights, particularly regarding fat distribution. Thus, an individual may present a BMI value within normal limits while simultaneously exhibiting central or visceral obesity. For this reason, multiple anthropometric indices, or even a strategic combination thereof, must be utilized in clinical diagnosis [8]. Because these anthropometric indices can be effectively deployed for identification, targeted intervention, or health impact evaluation, they have been designated as anthropometric health indicators [1]. A large study involving 177,792 participants suggests that, though remaining undetected in BMI-based screening, normal weight obesity increases the prevalence of cardiometabolic risk factors [9].

The weight-adjusted waist index (WWI), defined as waist circumference (WC) divided by the square root of body weight in kilograms, has been shown to be a strong predictor of several chronic diseases, including hypertension, diabetes, cardiovascular diseases, chronic kidney disease, and albuminuria, as well as a predictor of mortality [10,11,12]. WWI, as a promising new indicator, utilizes WC measurement and proves to be a more reliable predictive factor than BMI, especially in the case of central obesity [13]. An increase in WWI indicates a condition characterized by excessive accumulation of body fat and increased loss of muscle mass, which can directly and concretely assess central obesity [14].

The SAD-to-Waist Ratio combines two clinical measurements: the Sagittal Abdominal Diameter (SAD), which represents the anteroposterior diameter of the abdomen, and the Waist Circumference (WC). This metric is specifically utilized to estimate visceral adipose tissue—the intra-abdominal fat surrounding vital organs—which serves as a primary driver of cardiovascular disease and insulin resistance [15,16].

The precise evaluation of body composition and the early identification of metabolic risks represent both a major challenge and a critical priority in contemporary public health management. Among young populations, the transition from adolescence to young adulthood frequently coincides with the onset of university life. This critical developmental stage is characterized by drastic and often unfavorable changes in lifestyle habits.

Traditionally, screening for weight status and obesity-related risks has relied heavily on the Body Mass Index (BMI) [17,18]. Although its epidemiological utility remains undeniable due to its simplicity, its geometric and biological limitations are increasingly criticized in modern medical literature. BMI exhibits a structural inability to differentiate muscle mass from adipose tissue [19,20]. Furthermore, it fails entirely to reflect the regional distribution of body fat. From a pathophysiological perspective, metabolic dysfunction is driven not merely by total fat mass, but specifically by its accumulation within the abdominal region—namely ectopic and visceral fat—which is recognized for its highly pro-inflammatory secretory profile [17].

To overcome these limitations, cutting-edge research has shifted toward advanced anthropometric indices and three-dimensional mathematical volume models. Among these, the Adiposity Volume Index (AVI) and the Weight-Adjusted Waist Index (WWI) have demonstrated superior statistical accuracy in estimating intra-abdominal fat. While WWI successfully isolates waist circumference from the variability of total body weight, AVI provides a much more stable volumetric assessment of the trunk region. Concurrently, parameters such as total Body Surface Area (BSA) and sagittal abdominal diameter (reflected in the SAD-to-waist circumference ratio) complement the clinical picture, offering a refined geometric perspective on how the young organism reconfigures its somatic architecture without relying on expensive or irradiating imaging techniques. All these indices mentioned above are critical in defining an individual’s cardiometabolic risk. Waist girth and BMI are commonly used as markers of cardiometabolic risk [21]. Accumulating data however suggest that sagittal abdominal diameter (SAD) or “abdominal height” may be a better marker of intra-abdominal adiposity and cardiometabolic risk [21].

One non-invasive biomarker is the second-to-fourth digit ratio (2D:4D), which has been proposed as an indicator of prenatal androgen and estrogen exposure [22]. This ratio is established early in gestation and is reported to remain largely stable throughout life, suggesting its potential to bridge early developmental processes with physiological and pathological outcomes in adulthood [23,24]. Accordingly, the 2D:4D ratio has been linked to athletic performance, cognitive function, handedness, reproductive parameters, personality traits, and various cardiometabolic risk indicators [25].

The adult phenotype and the predisposition toward a specific pattern of adipose tissue distribution are not merely the result of current lifestyle choices; they exhibit a profound biological determinism established during the intrauterine period. In this context, the 2D:4D digit ratio serves as a stable anatomical marker that reflects the balance between fetal exposure to testosterone and estrogens during the first trimester of pregnancy. A lower 2D:4D ratio indicates a high prenatal androgen exposure, whereas a higher ratio is associated with a prominent estrogenic imprint [26,27].

Recent research suggests that this early hormonal programming modulates the subsequent sensitivity of receptors within regional adipose tissue [28]. For this reason, integrating a prenatal marker (R-2D:4D) alongside current anthropometric indices offers a unique opportunity to understand whether the metabolic vulnerability of young adults is a purely behavioral acquisition or if it possesses a pre-existing genetic and hormonal constitutional root, all achieved through a simple, non-invasive measurement of the index and ring fingers.

One of the fundamental pillars justifying the present study resides in the urgent need to develop assessment tools tailored to current socio-economic and psychological realities. In the contemporary medical context, large-scale monitoring of cardiometabolic risk through traditional clinical methods faces significant logistical and financial barriers. Although laboratory biochemical analyses represent the gold standard, they entail high operational costs, the consumption of medical supplies, and the necessity of a specialized infrastructure, making them difficult to implement as mass screening strategies within student communities. Furthermore, the psychological component of the young population plays an often underestimated role: a considerable percentage of young adults exhibit a profound aversion, fear, or even severe anxiety toward invasive medical procedures, particularly those involving venous blood sampling. This emotional barrier leads to the avoidance of preventive check-ups and the postponement of medical consultations, thereby masking latent risks.

Consequently, it becomes imperative to identify and validate entirely non-invasive, safe, rapid, and highly cost-effective methodologies that are universally accessible to any student, regardless of financial resources. Utilizing indices calculated strictly on mathematical foundations and surface measurements completely eliminates needle-related anxiety, demystifies the notion of restrictive medical check-ups, and allows for an excellent mapping of the cardiometabolic risk profile, thereby providing a sustainable and easily reproducible community screening solution.

Based on these premises, the primary purpose of the present research is to evaluate and cross-analyze the concurrent association and the diagnostic screening accuracy of ROC curves for classic and advanced anthropometric indices in identifying an elevated Adiposity Volume Index (AVI) among young university students within the studied cohort. By applying a stratified methodology, this study aims to examine how these diagnostic models vary, specialize, or alter their screening alignment under the combined influence of sex, specific academic program, and perceived stress levels (PSQ), providing a theoretical framework and a set of individualized critical cut-off thresholds capable of assisting in the early, non-invasive risk-assessment of metabolic vulnerabilities within this demographic, prior to the deployment of invasive procedures.

To achieve the general purpose, the following specific objectives were established: evaluating the diagnostic efficiency and determining the optimal cut-off thresholds for traditional structural somatometric indicators (BMI and BSA) in identifying the status of elevated adiposity; analyzing the screening capacity of the advanced Weight-Adjusted Waist Index (WWI) and determining its degree of statistical consistency across sex sub-cohorts; investigating the diagnostic value of secondary markers (the right-hand digital 2D:4D ratio and the sagittal abdominal diameter-to-waist circumference ratio) as constitutional correlates of regional adipose tissue accumulation; quantifying the concurrent relationship of perceived psychological stress (via the PSQ score) with metabolic status and determining its capacity for the clinical discrimination of visceral adiposity based on sex; and mapping how the curricular profile of university programs (Biomedical Sciences vs. Computer Science & Engineering vs. Social Sciences & Physiotherapy) modulates the concurrent interaction between psychological stress and somatometric markers.

### Research Hypotheses

In accordance with the proposed objectives and existing literature, the following working hypotheses were formulated:

**Hypothesis** **1 (H1). ** *Classic structural somatic indicators (BMI and BSA) maintain an important classification alignment and diagnostic accuracy in identifying elevated adiposity volume (defined by externally validated clinical AVI thresholds) across both sexes; however, the optimal cut-off thresholds exhibit significant variations between men and women*.

**Hypothesis** **2 (H2). ** *The advanced anthropometric index WWI (Weight-Adjusted Waist Index) presents a robust diagnostic value and a consistent screening performance across sexes, functioning as a stable concurrent covariate of abdominal adiposity within the studied cohort*.

**Hypothesis** **3 (H3). ** *The prenatal biological marker (R-2D:4D digit ratio) and regional geometric traceability (SAD-to-Waist Ratio) exhibit a pronounced sexual dimorphism in screening capacity, demonstrating a valid diagnostic performance specifically within the female student sub-cohort*.

**Hypothesis** **4 (H4). ** *The general level of perceived psychological stress (PSQ score) acts as a valid concurrent correlate for abdominal adiposity volume accumulation selectively, reaching statistical significance only within the female student cohort, reflecting a distinct sex-specific psychosomatic association under the pressure of the academic environment*.

**Hypothesis** **5 (H5). ** *The profile of the academic program followed by the students is linked to varied perceived stress levels and, consequently, co-varies with the statistical patterns of advanced anthropometric indices, generating structural differences among biomedical, technical, and social sciences specializations within the analyzed student groups*.

## 2. Materials and Methods

### 2.1. Participants

The total sample consisted of 445 university students from the University of Oradea, aged between 20 and 25 years. Participants were enrolled across three distinct academic programs: Biomedical Sciences (BS) (*n* = 144), Computer Science and Engineering (CSE) (*n* = 159), and Social Sciences and Physiotherapy (SSP) (*n* = 142). Regarding sex distribution, the cohort included 199 men (44.7%) and 246 women (55.3%). All subjects participated voluntarily and provided informed consent prior to data collection.

### 2.2. Psychosocial Measures

The Perceived Stress Questionnaire (PSQ): Psychological states and reactions resulting from confrontations with situations involving loss, threats, or hassles were identified using the Perceived Stress Questionnaire (PSQ), developed by Levenstein et al. in 1993 [29]. According to the authors, the questionnaire is a highly relevant instrument for establishing the level of perceived stress. The scale comprises 30 items describing potential emotional and mental reactions to demands that exceed an individual’s coping capacities, to understimulation, or to conflict situations. The participant’s task is to circle one of four response options (where the number 1 signifies “almost never” and the number 4 signifies “almost always”). For 8 of the 30 items, the scores provided by the proband are reversed. The total score, ranging from 30 to 120, allows for the classification of probands into one of three categories: low stress, moderate stress, and high stress [30]. Sample items include: “I feel frustrated and annoyed”, “I feel that too many demands are being made on me”, “I feel discouraged”, “I feel mentally exhausted”, and “I am full of worry about the future”.

### 2.3. Anthropometric Measurements and Working Procedure

To determine the target anthropometric indices (BMI, R-2D:4D Ratio, SAD-to-Waist Ratio, BSA, and WWI), the following direct structural measurements were performed: standing height (H), body weight (W), waist circumference (WC), sagittal abdominal diameter (SAD), second digit length (2D), and fourth digit length (4D).

Height was measured with an ADE wall taliometer with 1 mm precision (ADE^®^ GmbH, Hamburg, Germany) with individuals lightly dressed, without shoes, standing erect, back straight, heels together, and with feet slightly spread. Weight was measured with a digital device using bioelectrical impedance analysis (Omron BF-511; Omron Healthcare Co., Ltd., Kyoto, Japan).

WC was measured using a flexible, non-stretchable anthropometric tape. WC was recorded in centimeters at the midpoint between the lower border of the rib cage and the iliac crest along the midaxillary line, with participants standing erect and measured at the end of a normal expiration.

SAD was measured using a specialized sliding abdominal caliper (sliding anthropometer). The participant was placed in a supine position on a firm, flat examination table with knees comfortably bent at a 90° angle to relax the abdominal wall muscles. After a normal, quiet expiration, the investigator placed the fixed arm of the caliper beneath the lower back and lowered the sliding arm until it gently touched the highest point of the abdomen, typically at the level of the L4–L5 lumbar vertebrae or the natural umbilical line. The vertical depth was recorded to the nearest 0.1 cm.

The length of both the index finger (2D) and the ring finger (4D) was measured directly from the metacarpophalangeal joint (the basal crease where the finger meets the palm) to the most distal tip of the fleshy pad of the finger. The fingernail was strictly excluded from the measurement. The participant placed their right hand flat on a firm, level surface, palm facing upward, with the fingers fully extended, straight, and comfortably adducted. Measurements were executed manually using a professional YATO YT-7201 (Yato Tools Shanghai, China) stainless steel digital electronic caliper. This instrument features a linear capacitive measuring system with a high-definition LCD display, providing a resolution of 0.01 mm and an intrinsic accuracy of ±0.02 mm for measurements under 100 mm. The caliper jaws were applied gently against the anatomical landmarks to avoid skin or subcutaneous tissue compression that could artificially distort the data. Prior to taking any measurements, a comprehensive physical inspection was conducted. The investigator verified that the participant’s fingers were completely straight and entirely free of any physical deformities, congenital anomalies, joint inflammation, severe scarring, or previous orthopedic trauma that could alter the natural soft tissue profile or bone architecture.

Body Mass Index (BMI) is the official clinical indicator utilized to evaluate whether an adult presents a healthy weight relative to height. This index provides a straightforward and practical approach to classify weight status, where a high value (BMI ≥ 30 kg/m^2^) defines obesity—a state closely linked to multiple metabolic comorbidities and increased mortality rates [31,32]. The BMI metric is calculated by dividing the participant’s body weight in kilograms by the square of their body height measured in meters, according to the following mathematical formula:

$$BMI=\frac{Weight\;\left(kg\right)}{Height{\;\left(m\right)}^{2}}$$

The 2D:4D ratio (the length of the index finger divided by the ring finger) serves as a stable proxy biomarker of prenatal hormone exposure. A lower ratio (masculinized) indicates higher fetal testosterone, while a higher ratio (feminized) reflects higher prenatal estrogen. This ratio is established in the womb and remains stable throughout life [33]. The Right-Hand Second-to-Fourth Digit Ratio (R-2D:4D) is determined by dividing the exact length of the index finger by the exact length of the ring finger of the right hand. The calculation formula is as follows:

$$Right\;Hand\;2D:4D\;Ratio=\;\frac{2nd\;Digit\;Lenght\;(cm)}{4th\;Digit\;Lenght\;(cm)}$$

The Sagittal Abdominal Diameter-to-Waist circumference Ratio (SAD-to-Waist Ratio) combines a geometric depth parameter with a circumferential perimeter, offering a comprehensive three-dimensional perspective of visceral fat distribution. The SAD-to-Waist Ratio is calculated by dividing the sagittal abdominal diameter (measured in a supine position) by the standing waist circumference of the participant, according to the following mathematical formula:

$$SAD-to-WC\;Ratio=\;\frac{Sagittal\;Abdominal\;Diameter\;(cm)}{Waist\;Circumference\;(cm)}$$

Body Surface Area (BSA) is a calculated biometric parameter representing the total surface area of the human skin, expressed in square meters (m^2^). In clinical and epidemiological research, BSA serves as an essential indicator of metabolic mass and body size. The BSA is calculated by multiplying the participant’s height in centimeters by their weight in kilograms, dividing the product by 3600, and then extracting the square root of the resulting value, according to the following mathematical formula:

$$BSA=\;\sqrt{\frac{Height\;\left(cm\right)*Weight\;(kg)}{3600}}$$

The Weight-Adjusted Waist Index (WWI) evaluates central adiposity while minimizing the confounding effect of body weight. The WWI is obtained by dividing the participant’s waist circumference in centimeters by the square root of their body weight in kilograms, according to the following mathematical formula:

$$WWI=\;\frac{Waist\;Circumference\;(cm)}{\sqrt{Weight\;(kg)}}$$

### 2.4. Study Design and Procedure

This investigation was structured as a descriptive, cross-sectional study. Participants comprised university students from the University of Oradea, enrolled across three major academic programs, BS, CSE, and SSP, with ages ranging between 20 and 25 years.

Strict exclusion criteria were applied to ensure data integrity:

1. Enrolment in academic programs other than the three selected specializations;
2. Failure to state age or falling outside the designated 20–25 age bracket;
3. Failure to indicate biological sex;
4. Formally diagnosed chronic metabolic and/or cardiovascular diseases;
5. Non-completion or partial completion of the psychosocial scale.

Data collection was carried out through direct, face-to-face clinical and anthropometric interactions. Immediately following the completion of the physical measurements, each participant was provided with the printout containing the PSQ. To guarantee seamless data matching, the anthropometric recording sheets and the psychosocial questionnaire for each individual were physically stapled and coded together.

### 2.5. Ethical Considerations

This study strictly adhered to institutional and international research ethics guidelines. All subjects participated on a completely voluntary basis and were informed in advance regarding the scope, purposes, and design of the research.

To ensure absolute privacy, participants were explicitly instructed not to provide any identifying information, such as names or personal identification codes. The protocol emphasized that only biological sex and academic program affiliation were relevant to the target epidemiological analysis. All collected data were aggregated anonymously.

The study protocol was formally reviewed and approved by the Research Ethics Committee of the Faculty of Medicine and Pharmacy, University of Oradea. Furthermore, signed written informed consent was obtained from each individual participant prior to the initiation of any data collection or anthropometric measurement procedures.

### 2.6. Statistical Data Analysis

Statistical data analysis was performed using IBM SPSS Statistics software (Version 26.0; IBM Corp., Armonk, NY, USA). The statistical significance threshold was set a priori at *α* = 0.05, with *p* < 0.05 values considered statistically significant. The distribution and normality of continuous variables were preliminarily evaluated by analyzing skewness and kurtosis indices, with all values falling within the accepted limits for the use of parametric tests.

The data analysis strategy comprised the following successive methodological steps:

1. Frequency Analysis and Categorical Association: The distribution of participants by sex across the three academic programs (BS, CSE, and SSP) was evaluated using contingency tables (Crosstabs), and differences in proportions were tested using Pearson’s Chi-square test (χ^2^).
2. Descriptive and Comparative ANOVA Analysis: Anthropometric indicators, advanced adiposity indices, and the psychological variable were expressed as Mean ± Standard Deviation (Mean ± SD). To evaluate the differences among the means of the three academic programs, a One-Way ANOVA test was applied, strictly stratified by sex to control for inherent sexual dimorphism. Subsequent multiple comparisons were performed using the Tukey HSD post-hoc test.
3. Linear Association Analysis: Inter-correlation relationships between anthropometric predictors and perceived stress levels were evaluated by calculating Pearson correlation coefficients (r). For spatial efficiency and visual contrast, the matrix was stratified by sex using a diagonal split technique (data for the female group are presented below the main diagonal, while data for the male group are presented above the diagonal).
4. Predictive Modeling via Multiple Linear Regression: Multiple linear regression models (Enter method) were constructed with AVI as the criterion (dependent) variable to evaluate the predictive relationships among compositional indices (BMI, BSA, WWI), the biological marker (the 2D:4D digit ratio), the distribution indicator (the SAD-to-Waist Ratio), and the global stress score obtained from the PSQ. The analysis evolved from a general perspective to one of high specificity: first globally by sex, subsequently stratified by academic program, and finally through an advanced three-dimensional approach (sex × academic program × binary stress status). For each model, the coefficient of determination (*R*^2^), the *F*-test for model fit, unstandardized coefficients (*B)*, standard errors (SE), standardized coefficients (*β*), and t-values were reported. Collinearity diagnostics were verified using the Variance Inflation Factor (VIF), with the obtained values falling below the critical threshold of 5.0, confirming the absence of multicollinearity among the predictors.
5. Predictive Performance Analysis and Cut-off Threshold Estimation (ROC Analysis): The discriminatory capacity of the indices (BMI, R-2D:4D, SAD-to-Waist Ratio, BSA, WWI) and the PSQ was evaluated through Receiver Operating Characteristic (ROC) curve analysis. Global performance was quantified by the Area Under the Curve (*AUC*) with 95% Confidence Intervals (CI). Optimized critical cut-off thresholds were determined by maximizing the Youden index, reporting the associated sensitivity, specificity, as well as positive and negative predictive values.

## 3. Results

### 3.1. Descriptive Statistics and Distribution Analysis

In alignment with the research objectives, the preliminary investigation assessed the sample distribution across sexes and academic programs (Table 1).

Analysis of participant distribution by sex and academic program (Table 1) revealed major statistically significant differences among the three studied groups (*χ*^2^ (2) = 45.718, *p* < 0.001). Within the Biomedical program, a clear predominance of the female population was recorded (74.3% women vs. 25.7% men). In direct contrast, the Computer Science and Engineering profile was heavily dominated by men (64.2% men vs. 35.8% women). The sex distribution was relatively more balanced within the Social Sciences and Physiotherapy program, although a slight premium was maintained in favor of female students (57.7% women vs. 42.3% men). Overall, the total sample (*n* = 445) was composed of a relatively homogeneous structure, with a slight prevalence of the female sex (55.3% women vs. 44.7% men).

### 3.2. Comparative Analysis of Anthropometric Health Indicators

The comparative One-Way ANOVA, stratified by sex (Table 2), revealed specific significant variations in anthropometric parameters across the studied academic programs. Regarding BMI, clear differences were recorded in both men (*F* = 6.418, *p* = 0.002) and women (*F* = 3.58, *p* = 0.031), with students from the BS program presenting the highest values. The post-hoc (Tukey) analysis confirmed that men from BS had a significantly higher BMI than their peers from CSE and SSP, whereas women from BS significantly exceeded only the female students from CSE. In terms of advanced anthropometric indices, the SAD-to-waist ratio exhibited significant variations exclusively within the female sample (*F* = 4.332, *p* = 0.014), where women from BS recorded significantly lower values compared to those from CSE. Conversely, the BSA (*F* = 4.115, *p* = 0.018) and WWI (*F* = 3.696, *p* = 0.027) indicators showed a significant dynamic only among men, with BS students displaying significantly higher values than those from SSP. The R-2D:4D ratio (a stable prenatal marker) and the overall perceived stress level did not present statistically significant differences across academic programs, regardless of sex (*p* > 0.05). However, at the descriptive level, a general trend toward amplified stress scores was observed within the female population compared to the male population across all study programs.

### 3.3. Anthropometric and Psychosocial Parameters Co-Varying with the Abdominal Volume Index (AVI) Within the Studied Student Cohorts

The inter-variable correlation analysis, stratified by sex (Table 3), indicated distinct patterns of association between anthropometric parameters and the psychological component. Within the female population, a positive, weak-to-moderate, but highly statistically significant correlation was observed between BMI and the PSQ score (*r* = 0.182, *p* < 0.01). Remarkably, this association was entirely absent in the male sample (*r* = −0.045, *p* > 0.05), suggesting a sex-differentiated psychosomatic reactivity. Classic and advanced anthropometric indicators (BMI, BSA, WWI, and the SAD-to-Waist Ratio) exhibited strong mutual positive correlations in both subgroups (*p* < 0.01), with the closest link recorded between BMI and BSA (in women: *r* = 0.842; in men: *r* = 0.736). Furthermore, the prenatal marker represented by the 2D:4D digit ratio manifested significant association patterns with current body composition parameters. In women, a higher R-2D:4D ratio was significantly associated with higher values of BMI (*r* = 0.449), BSA (*r* = 0.329), and WWI (*r* = 0.286), all at a threshold of *p* < 0.001. In men, although the associations remained significant, their intensity was visibly lower (with WWI: *r* = 0.318, *p* < 0.01; with BMI: *r* = 0.238, *p* < 0.01; with BSA: *r* = 0.170, *p* < 0.05).

As demonstrated in Table 4, statistically significant differences emerged between the estimates generated by the regression equations and the mean outcomes for the male cohort (F(6, 199) = 2257.066, *p* < 0.001) and the female cohort (*F*(6, 246) = 31.311, *p* < 0.001). The proportion of variance in abdominal volume status (the coefficient of multiple determination) explained by the joint action of BMI, R-2D:4D, SAD-to-Waist Ratio, BSA, WWI, and PSQ yielded an *R*^2^ = 0.986 for the male students, indicating that these variables account for 98.6% of AVI variance. In the female cohort, the multiple coefficient of determination (*R*^2^ = 0.440) demonstrated that the predictors contribute 44.0% to AVI variance, with the effect being moderate-to-strong in this case. Overall, the implemented model indicates a powerful and substantial impact of the selected predictors on AVI. The utilized models highlight the critical importance of combining anthropometric predictors with genetic (R-2D:4D) and psychological factors, thereby enabling excellent accuracy in identifying the risk of central fat accumulation among young adults.

Multiple linear regression models run separately by sex (Table 5) revealed major structural differences in the determinism of the AVI. Within the male sample, the global model displayed an extremely high predictive capacity (*R*^2^ = 0.986, *F* = 2257.066, *p* < 0.001), with the variance of AVI being explained almost entirely by the combined set of predictors. The strongest independent positive covariates were the WWI (*β* = 0.411, *p* < 0.001), BSA (*β* = 0.393, *p* < 0.001), and BMI (*β* = 0.347, *p* < 0.001) indicators, followed by a modest but significant contribution from the SAD-to-Waist Ratio (*β* = 0.024, *p* = 0.011). The R-2D:4D ratio and perceived stress did not influence the male model. In contrast, within the female sample, the model explained 44.0% of the AVI variance (*R*^2^ = 0.440, *F* = 31.311, *p* < 0.001). The strongest positive association was observed for BMI (*β* = 0.362, *p* = 0.001), followed by WWI (*β* = 0.251, *p* < 0.001) and BSA (*β* = 0.228, *p* = 0.013). Remarkably, unlike in men, the 2D:4D digit ratio emerged as a significant negative independent correlate among women (*B* = −23.237, *β* = −0.112, *p* = 0.043), indicating a link between the prenatal hormonal profile and adult adiposity volume in young adulthood. The SAD-to-Waist Ratio and perceived stress levels did not manifest significant concurrent associations effects (*p* > 0.05) in the female model.

The male global cohort model generated a substantial effect size (f^2^ = 70.429), while the female global cohort model similarly demonstrated a robust effect size (f^2^ = 0.786). Consequently, both global regression configurations achieved the maximum theoretical statistical power of 1.000 (100%), verifying that the baseline sample sizes provide more than adequate safety against Type II statistical errors.

Simultaneous stratification of the predictive equations based on sex and academic program (Table 6) revealed a profound structural heterogeneity within the female population. While the predictive models for men remained extremely stable and powerful across all three university profiles (*R*^2^ ranging between 0.986 and 0.989, *p* < 0.001), driven massively by the triad of BMI, BSA, and WWI, the female subgroup experienced major variations. Thus, for female students in the BS program, the explanatory capacity of the model decreased dramatically to only 29.6% (*R*^2^ = 0.296, *F* = 7.018, *p* < 0.001). Within this specific group, the only independent predictor with a statistically significant impact on AVI was the WWI (*B* = 1.420, *β* = 0.218, *p* = 0.023), whereas classic markers such as BMI (*p* = 0.093) lost their direct significance. In contrast, for female students in the CSE (*R*^2^ = 0.976) and SSP (*R*^2^ = 0.991) programs, the predictive architecture realigned with the male pattern, being sovereignly and highly significantly (*p* < 0.001) dominated by the same heavy anthropometric parameters (BMI, BSA, and WWI). Among men, a notable particularity was identified in the BS profile, where the perceived stress score emerged as a significant independent negative predictor (*B* = −0.020, *p* = 0.007), an effect that was completely absent in the other specializations.

All stratified subgroups presented significant effect sizes according to Cohen’s thresholds, spanning from (f^2^ = 0.420) (in the female biomedical sample) to (f^2^ = 110.111) (in the female social sciences profile). Driven by these intense effect dynamics, every individual sub-model achieved an ideal observed power of 1.000 (100%). This maximum value confirms that the sample size within each academic program provides full statistical security and protects the regression findings against overfitting.

Multiple linear regression analysis performed by stratifying the sample based on sex and the presence of stress (Table 7) highlighted a particularly powerful psychosomatic interaction phenomenon manifested exclusively within the female group. Within the male group, the behavior of the multi-variable association model remained highly stable and unaffected by variations in adaptive states. In both men with low stress levels (*R*^2^ = 0.987, *F* = 1675.423, *p* < 0.001) and those with high stress levels (*R*^2^ = 0.986, *F* = 1063.202, *p* < 0.001), the variance of AVI was explained almost entirely by the classic anthropometric triad consisting of BMI, BSA, and WWI, with all variables maintaining a highly significant contribution (*p* < 0.001). In distinct contrast, within the female sub-cohort, the presence of distress is associated with a different pattern of multi-variable co-variation. In the absence of stress (0.00 score), the anthropometric parameters account for 88.3% of the variance in the AVI (*R*^2^ = 0.883, *F* = 145.320, *p* < 0.001), with BMI exhibiting the highest standardized coefficient (*β* = 0.645, *p* < 0.001). However, under conditions of psychological stress (1.00 score), the explanatory capacity of the model shifts to 41.3% (*R*^2^ = 0.413, *F* = 19.391, *p* < 0.001). Notably, BMI does not maintain its independent statistical link (*p* = 0.264). In this state of distress, the female equation shows an increased statistical weight for the WWI compositional index (*β* = 0.396, *p* < 0.001), and the prenatal biological marker represented by the 2D:4D digit ratio emerges as a significant negative independent covariate (*B* = −45.463, *β* = −0.186, *p* = 0.015).

The resulting effect values prove highly robust, with the lowest indicator identified in the low-stress female group (f^2^ = 7.547) and the peak value in the low-stress male sample (f^2^ = 75.923). Owing to these strong associative dynamics, all four configurations reached an absolute observed power of 1.000 (100%), mathematically confirming the stability of the regression parameters against sample size fluctuations.

Three-dimensional multiple linear regression analysis of the sample (sex × academic program × stress status) (Table 8) indicated that the variation in anthropometric association observed within the female cohort is not a generalized phenomenon; rather, it is a profoundly specific effect, isolated exclusively among female students in the BS program (*R*^2^ = 0.313). In the absence of psychological stress (0.00 score), the model excellently explains 90.8% of the variance in AVI for BS women, with the equation classically controlled by BMI (*β* = 0.400, *p* = 0.001), BSA (*β* = 0.360, *p* = 0.001), and, in a positive manner, by the prenatal marker represented by the 2D:4D digit ratio (*β* = 0.184, *p* = 0.007). However, upon the onset of stress (1.00 score), the anthropometric model collapses dramatically, and the heavy somatic parameters (BMI and BSA) completely lose their statistical significance (*p* > 0.05). In this specific context of distress, the female abdominal volume index exhibits a significant linear association with the WWI compositional index (*β* = 0.428, *p* = 0.002) and with the 2D:4D ratio, which shows an inverse direction in its coefficient, acting as an independent negative covariate (*B* = −87.780, *β* = −0.304, *p* = 0.019). In contrast to this atypical dynamic in BS, female students in the CSE (*R*^2^ > 0.97) and SSP (*R*^2^ > 0.97) profiles maintain strong structural stability in their models, with the triad of BMI, BSA, and WWI maintaining a consistent linear association regardless of the presence or absence of stress (*p* < 0.05). Among the male cohort, a substantial uniformity was observed across all academic programs (*R*^2^ constantly ranging between 0.978 and 0.989, *p* < 0.001). In men, the model displays a consistent configuration defined by the same triad (BMI, BSA, and WWI), with the only micro-variation identified among BS students presenting low stress levels, where the SAD-to-Waist Ratio manifested a marginal but significant positive influence (*B* = 15.303, *p* = 0.047), an effect that dissipates completely under the action of stress.

To confirm that the stratified subgroup regression analyses were not subject to overfitting or type II errors, a post-hoc power analysis was performed for all sub-models using Cohen’s (f^2^) effect size calculation, with the results detailed (see Table 8).

The sex-stratified receiver operating characteristic (*ROC*) analysis identified distinct parameters of association and classification thresholds for both anthropometric covariates and bio-psychosocial markers in relation to an elevated AVI status (Table 9).

**Figure 1:**
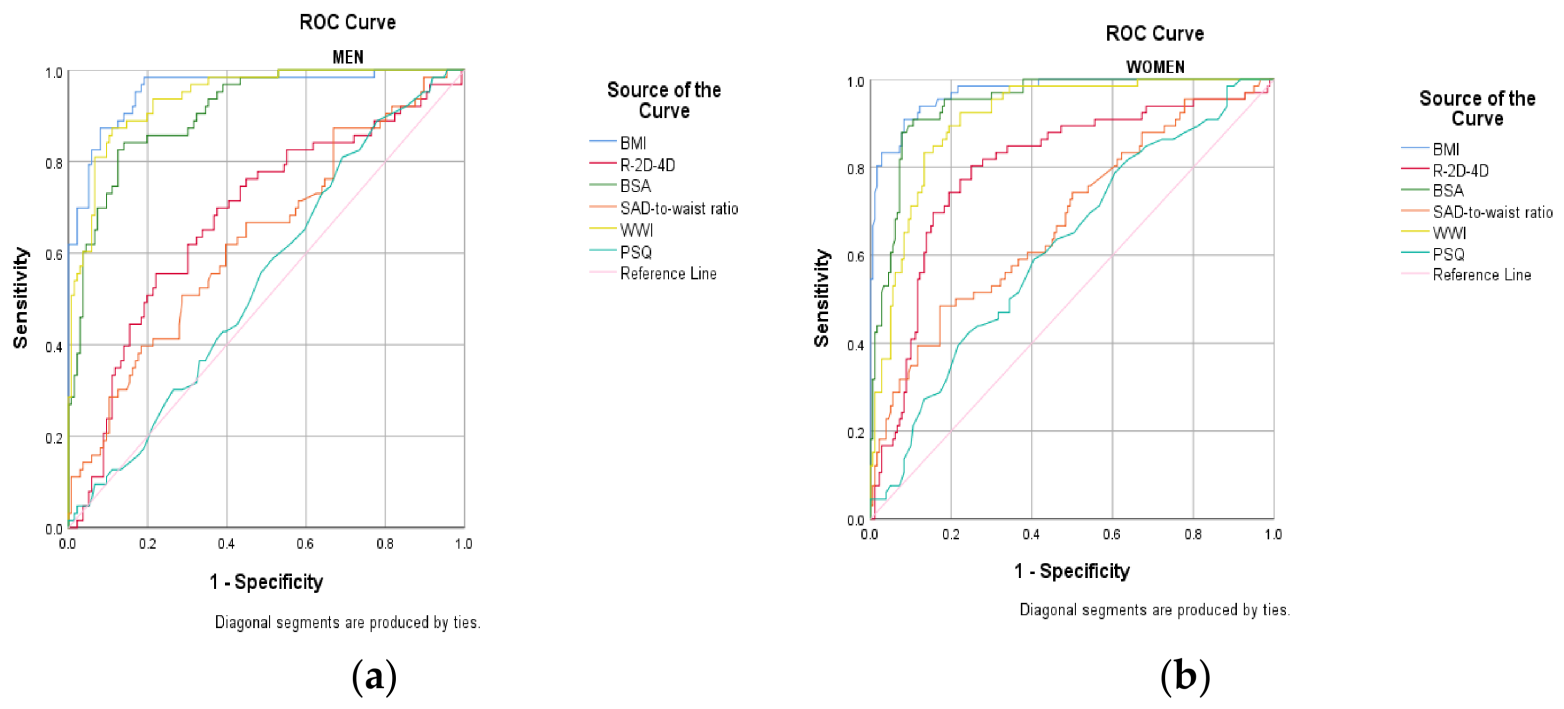
Receiver operating characteristic (ROC) curves for abdominal volume. BMI = Body Mass Index; R-2D:4D = Right Hand 2D:4D Ratio; SAD-to-Waist Ratio = Sagittal Abdominal Diameter to Waist Circumference Ratio; BSA = Body Surface Area; WWI = Weight-Adjusted Waist Index; PSQ = Perceived Stress Questionnaire. (**a**). In male group; (**b**). in female group.

Among male students (Table 9, Figure 1a), traditional anthropometric variables showed high classification alignment, with BMI reaching an *AUC* of 0.957 (95% *CI*: 0.928–0.987, *p* < 0.001) at an optimal cut-off threshold of 26.96 kg/m^2^ (Sensitivity = 87.3%, Specificity = 91.9%). WWI also demonstrated a tight statistical linkage (*AUC* = 0.941, 95% *CI*: 0.909–0.972, *p* < 0.001) at an optimal cut-off threshold of 10.12. Concurrently, the prenatal biological marker (R-2D:4D ratio) exhibited a statistically significant concurrent association with elevated AVI (*AUC* = 0.680, 95% *CI*: 0.598–0.762, *p* < 0.001), yielding an optimal cut-off value of 0.9945 (Sensitivity = 55.6%, Specificity = 77.9%). Conversely, perceived stress scores did not achieve a statistically significant concurrent link within the male student subgroup (*AUC* = 0.545, 95% *CI*: 0.462–0.628, *p* = 0.306).

For female students (Table 9, Figure 1b), indicators of association were consistently observed across all dimensions. Traditional parameters exhibited high structural alignment, led by BMI (*AUC* = 0.972, 95% *CI*: 0.953–0.991, *p* < 0.001, Cut-off = 24.89 kg/m^2^, Sensitivity = 90.9%, Specificity = 91.7%). Notably, the bio-psychosocial markers displayed stronger patterns of association in female students compared to male students. The R-2D:4D ratio demonstrated high discriminatory capacity (*AUC* = 0.797, 95% *CI*: 0.731–0.864, *p* < 0.001) at an optimal threshold of 1.0077 (Sensitivity = 80.3%, Specificity = 75.0%). Furthermore, perceived stress was confirmed as a statistically significant concurrent correlate of elevated abdominal status in female students (*AUC* = 0.621, 95% *CI*: 0.543–0.698, *p* = 0.004), identifying an optimal classification threshold at a score of 65.50 (Sensitivity = 63.6%, Specificity = 53.9%).

## 4. Discussion

The results indicate a sex-based segregation across study profiles (a female predominance in BS and a male majority in CSE), but a balanced overall distribution (44.7% men vs. 55.3% women). This structure, showing a significant difference (*p* < 0.001), allows for a nuanced, sex-controlled analysis of the selected anthropometric indicators and perceived stress, effectively accounting for the inherent sexual dimorphism.

Furthermore, our findings highlight an interesting asymmetry between classic and advanced anthropometric indicators. The fact that students in the BS program (particularly men) present higher BMI and WWI scores compared to their counterparts in other specializations may reflect specific lifestyle characteristics or dietary behaviors characteristic of the initial years of university life in the medical field—a phenomenon partially documented in the literature [33,34,35,36]. On the other hand, the lack of statistical significance for the R-2D:4D ratio across academic programs validates its nature as a biologically fixed prenatal marker, independent of subsequent vocational or academic trajectories, while preserving typical sexual dimorphism (lower values in men, typically below 1.00). Similarly, the uniformity of perceived stress across academic profiles, combined with consistently higher scores in women, underscores that psychological vulnerability within the university environment is mediated by sex-based factors rather than the curricular specificities of the chosen program.

The sexual dimorphism observed in correlation dynamics provides additional evidence regarding the biological and behavioral determinism of weight status. The female-exclusive correlation between the perceived stress score and BMI supports hypotheses in medical psychology regarding sex-differentiated coping mechanisms; young women tend to associate emotional distress with alterations in dietary behavior (such as emotional eating), leading over time to increased body mass, a phenomenon less pronounced in men at this age stage. Furthermore, the stronger association of the 2D:4D digit ratio with adiposity indicators (BMI, WWI) in women compared to men suggests that the prenatal hormonal environment (a higher ratio indicating lower fetal testosterone and higher estrogen exposure) may reflect an underlying biological link associated with the variations in adipose tissue accumulation in young adulthood, manifesting more prominently among female students.

A finding of particular scientific interest in our study is represented by the divergent association profile of the right-hand 2D:4D digit ratio. The fact that this prenatal biological marker demonstrated a significant negative association on AVI exclusively among female students (*p* = 0.043) suggests a potential link between early prenatal endocrine indicators and adult regional body volume distribution within the studied cohort. A lower R-2D:4D ratio, theoretically associated with increased exposure to fetal testosterone, appears to act as a long-term vulnerability factor for adipose tissue accumulation in women—a dynamic that is completely masked when the analysis is performed without sex stratification. Furthermore, the variation between the coefficient of determination in men (*R*^2^ = 0.986) and that in women (*R*^2^ = 0.440) suggests that the geometric configuration of body volume evaluated by AVI displays a high structural alignment with total body mass and surface area (BSA, BMI) within the male sub-cohort. In women, however, AVI displays a different pattern of variability within the studied sub-cohort, suggesting that non-anthropometric parameters may account for part of the variance that is not fully captured by standard parameters. The collapse of the coefficient of determination (*R*^2^ = 0.296) observed exclusively among female students in the BS program represents an important element of novelty. This suggests that in the case of young women pursuing rigorous medical studies, AVI ceases to be a simple linear reflection of overall body mass (BMI), being governed instead by a far more complex dynamic. The fact that the WWI maintains a consistent linear association within this cohort suggests its potential utility for non-invasive risk screening. WWI succeeds in capturing subtle variations in abdominal fat distribution independently of gross body weight, offering a much higher screening accuracy for this specific sample category. Concurrently, the statistically significant negative coefficient for perceived stress within the male biomedical student cohort (*p* = 0.007) indicates a distinct pattern of association: under conditions of high academic distress [37], lower values of the abdominal volume index are observed within this sub-cohort, in contrast to the behaviors described in women, further underscoring the necessity for sex-individualized approaches in clinical assessment [38,39].

This statistical variation in the multi-variable model alignment under the conditions of stress within the female cohort (*R*^2^ decreasing from 88.3% to 41.3%) provides localized descriptive data regarding the co-variation in psychosocial and physical parameters among university students. The complete loss of BMI’s significance as a weight-related predictor under stress conditions (*p* = 0.264) demonstrates that, under the influence of psychological distress, adiposity accumulation or volume in young women no longer follows a linear or homogeneous weight increase model. Pathophysiologically, chronic stress activates the hypothalamic–pituitary–adrenal (HPA) axis, generating a hypersecretion of cortisol. Cortisol, due to the high density of glucocorticoid receptors in the mesenteric region, promotes the redistribution of deep adipose tissue and alters body volume geometry in a manner that classic BMI cannot capture [40]. The major relevance of the modern WWI (*p* < 0.001) under stress conditions strengthens this hypothesis, given that WWI adjusts waist circumference relative to total body weight, thereby isolating metabolically induced central-adipose accumulation. Furthermore, the significant negative association of the 2D:4D digit ratio as a negative predictor (*p* = 0.015) exclusively under stress conditions suggests that the prenatal hormonal indicators may co-vary with adult regional body volume distribution within this specific cohort. This biological marker co-varies with the constitutional sensitivity of tissues to stress in adulthood, governing the pattern of fat storage when the organism’s homeostasis is psychologically disrupted—a dynamic that is completely absent in the male sample.

This advanced three-dimensional exploration validates a crucial hypothesis: the interaction between the adaptive psychological state (stress) and anthropometric indicators is critically mediated by the vocational-academic environment in young women. The observation that the variation in the multi-variable model alignment occurs primarily among stressed female students in the BS program suggests that this sub-cohort displays a distinct pattern of bio-psychosocial covariation within the studied sample. Medical and biomedical education imposes chronic stress patterns characterized by high performance pressure and sleep deprivation, factors recognized for triggering severe dysfunction of the hypothalamic–pituitary–adrenal (HPA) axis. The resulting pulsatile and chaotic secretion of cortisol acts selectively, inhibiting peripheral storage and promoting deep central-abdominal fat accumulation [41]. This phenomenon explains why BMI and BSA (indicators of overall body mass) does not demonstrate a statistically significant linear association with AVI among high-stress female medical students (*p* > 0.05), leaving WWI as the sole valid compositional indicator (*p* = 0.002). Biologically, the association profile of the R-2D:4D digit ratio within this sub-cohort is distinct. In female students with low stress levels, R-2D:4D displays a positive coefficient, reflecting the standard female dimorphic pattern where prenatal estrogen exposure favors normal gynoid adiposity. Under the effect of stress, however, the reversal of the coefficient to a negative direction (*B* = −87.780, *p* = 0.019) demonstrates that women with a lower R-2D:4D ratio (increased prenatal testosterone exposure, indicating a more androgenic biometric profile) become hyper-reactive to cortisol, developing a much more pronounced central adiposity volume. These prenatal hormonal indicators may potentially co-vary with adult regional body volume distribution under conditions of higher perceived academic stress within the studied female biomedical cohort. This statistical pattern was not observed within the technical or social sciences cohorts, where coping mechanisms or the nature of curricular stressors may take different forms [42].

The sex-stratified analysis using receiver operating characteristic (ROC) curves provided an empirical basis for evaluating the concurrent association between various parameters and an elevated Abdominal Volume Index (AVI). The observed classification profiles vary according to both biological sex and the nature of the analyzed markers.

The elevated area under the curve (*AUC*) values obtained for traditional anthropometric parameters, specifically BMI (0.957 in men, 0.972 in women), confirm a tight mathematical and structural alignment with AVI. Given the cross-sectional design of the present study, these data do not imply a causal pathway. Instead, they demonstrate that these traditional parameters and AVI co-vary simultaneously in terms of physical mass and body surface area. Within a multivariable framework, incorporating these traditional parameters is methodologically necessary to serve as anthropometric covariates. Controlling for these general physical dimensions ensures that the observed associations for the non-anthropometric markers remain independent and non-confounded.

In this context, the prenatal biological marker, represented by the R-2D:4D ratio, demonstrated an independent statistical association with an elevated AVI status in both subgroups, though the classification accuracy was more pronounced among female students (*AUC* = 0.797) compared to male students (*AUC* = 0.680), aligning with known dimorphic anatomical baselines. These findings suggest that prenatal hormonal exposure patterns, as reflected by digit ratios, maintain a concurrent statistical link with adult regional fat distribution, independent of concurrent general body mass or surface area.

Furthermore, psychological markers displayed distinct, sex-specific patterns of covariation. Perceived stress demonstrated a statistically significant concurrent association with an elevated AVI exclusively among female students (*AUC* = 0.621, *p* = 0.004), with an optimal classification threshold identified at a score of 65.50. In contrast, no statistically significant association was observed between perceived stress and AVI status within the male student subgroup (*AUC* = 0.545, *p* = 0.306). These outcomes suggest that psychological distress and regional body volume distribution are interrelated in a sex-dependent manner, highlighting the necessity of stratified epidemiological approaches when analyzing psychosocial and anthropometric indicators simultaneously. The identification of this value as the optimal risk threshold for perceived stress within the female subgroup provides clinicians with a clear psychometric screening indicator: exceeding this score is associated with an increased probability of accelerated visceral fat accumulation—an effect likely mediated through the neuroendocrine pathways of the HPA axis and glucocorticoid secretion [43]. Furthermore, the solid performance of the R-2D:4D digit ratio in women demonstrates that the constitutional sensitivity of fatty tissues and the geometric pattern of storage in young adulthood are under strong latent intrauterine hormonal programming. High estrogen exposure during prenatal development is significantly related to the development of excessive body weight in men and women and the accumulation of subcutaneous fat in the arms, thighs, and lower legs in women with obesity. This relationship indicates a new area of activity in the field of obesity prevention [25]. Moreover, it seems that the 2D:4D index (especially of the right hand) may serve as an early indicator of metabolic vulnerabilities in humans [44]. Additionally, the 2D:4D ratio in general, but particularly that of the right hand, serves as a very good biomarker for metabolic syndrome and cardiovascular risk [25]. In men, the strict isolation of diagnostic capacity solely within pure somatometric parameters (BMI, BSA, WWI) and the clinical failure of adaptive indicators reconfirm that the male body status is significantly more conservative biologically, being governed at this stage of life by raw geometric laws directly related to total surface area and body mass, a phenomenon also noted in other studies [45,46].

This major sex-based discrepancy can be grounded in two interdependent mechanisms: one neuroendocrine and one behavioral in nature [47].

The most widely utilized method for screening weight status and obesity-associated risk has traditionally relied on body mass index (BMI) [17,18]. Previous evidence suggests that BMI alone may not adequately identify young adults at increased cardiometabolic risk, as individuals with normal body weight may still exhibit an unfavorable body composition profile. Studies have shown that comprehensive body composition assessment provides clinically relevant information beyond conventional anthropometric indices and improves the identification of individuals with increased metabolic risk. Furthermore, sex-specific differences, together with lifestyle-related factors such as nutritional habits, sleep patterns, and physical activity, should be considered when evaluating body composition and cardiometabolic health in young adults [48,49]. These findings support the use of complementary anthropometric and body composition measures rather than relying solely on BMI. Thus, although its epidemiological utility is indisputable due to its ease of calculation, its geometric and biological limitations are increasingly criticized in modern medical literature. Studies have reported that BMI suffers from a structural inability to distinguish between muscle mass and adipose tissue [20]. Furthermore, it completely fails to reflect the regional distribution of body fat. From a pathophysiological perspective, it is not merely total fat mass that drives metabolic dysfunction, but rather its abdominal localization (ectopic and visceral fat)—which is known to possess a highly pro-inflammatory secretory profile [17].

Thus, while its epidemiological utility remains indisputable due to its ease of calculation, the index’s geometric and biological limitations are increasingly criticized in modern medical literature. Studies have reported that BMI suffers from a structural inability to distinguish between muscle mass and adipose tissue [20]. Furthermore, it fails to reflect the regional distribution of body fat. From a pathophysiological perspective, metabolic dysfunction is driven not merely by total fat mass, but rather by its abdominal localization (ectopic and visceral fat), which is known to possess a highly pro-inflammatory secretory profile [17].

Given the aforementioned studies, the scientific community has recently characterized a highly significant and clinically relevant phenotype: normal-weight obesity. Normal-weight obesity defines individuals who present with a normal body mass index (BMI) yet exhibit underlying metabolic complications, such as insulin resistance, hypertension, and dyslipidemia. Despite maintaining a normal body weight, these individuals harbor concealed visceral fat deposits surrounding internal organs.

However, limited data exist regarding the cardiometabolic profile of individuals who are non-obese according to body mass index (BMI) yet exhibit a high body fat percentage (BFP)—a phenotype frequently observed within the Indian population [50]. The authors examined both the prevalence of normal-weight obesity (NWO) and the cardiometabolic profile of individuals with NWO who were at high risk for type 2 diabetes mellitus (T2DM) within a South Asian population; they noted that nearly one-third of this high-risk cohort presented with normal-weight obesity. Furthermore, they highlighted that the significantly higher cardiometabolic risk associated with elevated adiposity, even in individuals with a low BMI, carries profound implications for the identification and management of this condition in clinical practice.

Based on their study in children, Farooqui et al. (2026) suggested that normal-weight obesity (NWO) represents a clinically relevant yet underrecognized pediatric phenotype, emphasizing that an exclusive reliance on body mass index (BMI) may delay the identification of vulnerable children [51]. Similarly, in a recent study targeting young adults, Falbova et al. (2025) observed that NWO is relatively prevalent among this population and is associated with a greater visceral fat area alongside reduced lean body mass, despite a normal BMI [48]. These findings underscore the importance of assessing parameters beyond BMI when evaluating health risks in young adults. Furthermore, this phenomenon extends beyond younger cohorts, as recent literature also provides evidence of this condition within the general adult population [8].

From a behavioral and psychometric perspective, young female students exposed to high levels of stress tend to adopt coping strategies radically different from those of men, frequently oriented toward dysfunctional dietary habits known as “emotional eating” [38,52]. Under the pressure of stress, women present a statistically more pronounced tendency to consume hypercaloric foods rich in refined carbohydrates and saturated fats—a mechanism that offers a temporary reduction in anxiety by stimulating dopaminergic reward pathways [53]. Correlating this behavior with the physical inactivity specific to exam periods explains why the threshold of 65.50 points on the PSQ isolates the risk of increased AVI with such high accuracy exclusively within the female sample.

An interesting 2020 study showed that AVI is higher in people with depression/anxiety [54]. AVI takes place among the best indicators of abdominal fat deposition [55]. The utilization and combination of anthropometric indices in identifying central or visceral obesity, especially in individuals presenting a normal weight, is of paramount importance; we share the perspective of de Jesus et al. [8], who obtained unfavorable cardiometabolic marker values in individuals with normal-weight obesity compared to normal-weight individuals who did not exhibit central or visceral obesity—a phenomenon also reported in other studies [17]. Previous research [56] has demonstrated the existence of differences in stress reactions based on various demographic variables. Thus, women and older individuals exhibit more stress reactions, likely due to a more pronounced tendency to appraise many life situations as threatening and to utilize emotional coping strategies [57]. Low socio-economic status is associated with an increased level of distress in men. Furthermore, professional roles are related to stress in married women with children who experience low levels of cooperation between partners [58].

Studies such as those conducted by Barnett et al. [59] show that female students manifest a higher level of self-criticism in response to hypothetical failures than male students, suggesting that women may perceive threatening events as more stressful than men do. More recent research [60,61] has uncovered a strong link between negative life events and suicidal ideation or attempts. This relationship was moderated by a sense of competence, autonomy, and the satisfaction of basic psychological needs.

On the other hand, Szabo and Marian [62] found that university students face pressures related to both academic performance and family-related stressors (such as financial situations, ill partners, or children). Social support was identified as a critical mitigating factor against the stress experienced by students.

Performance expectations, in turn, determine a series of stress consequences, manifested as physiological, emotional, social, cognitive, and performance-related stress. Research conducted on the Romanian university student population has also found that a relationship exists among active stress management (taking active steps to try to eliminate the stressor), the positive reinterpretation of stress, and personality variables. Other authors emphasize that students’ sense of control over a stressful situation can have a mitigating effect [63]. Female students appear to appraise stressful situations in a more negative manner. Additionally, male students report a higher level of leisure-time satisfaction than female students. However, studies also show that some individuals resort to negative coping strategies to manage stressors—such as self-criticism, alcohol, drugs, and internet addiction—which are not helpful in the long term [39].

Perceived stress, coping strategies, and social support contribute significantly to students’ well-being. In the same vein, Sandler [64], who studied perceived stress and academic performance among university students, argues that higher education institutions must provide relevant curricula and services tailored to students’ needs. Many students attempt to complete their studies and workplace tasks simultaneously, in addition to managing family responsibilities, which frequently generates supplementary stress.

From the analysis of the literature, two important conclusions emerge: first, it is essential to understand the specific sources of stressors faced by university students, and second, it is crucial to identify the various reactions to these stressors.

Abdominal obesity, whether central or visceral, is clearly a major risk factor for metabolic syndrome. Therefore, a careful and thorough approach is imperatively needed in the prevention, identification, and appropriate treatment of obesity. Diagnosis is achieved based on anthropometric indices associated (either directly or indirectly) with adiposity and its distribution within the body, as well as through the combination of these indices [65]. Combining anthropometric indices alone, or combining them with digit ratios (which represent secondary anthropometric markers), as well as with psychological factors, can provide a more comprehensive picture of cardiometabolic risk.

The fundamental originality and innovative character of our study reside in its holistic and three-dimensional approach to the interaction among biology, academic profile, and psychological state. Beyond strictly somatometric determinism, psychological stress represents a major systemic modulator of energy metabolism.

The results of our research underscore the urgent need to reconfigure preventive medicine programs within the university environment. They demonstrate that weight and adiposity management in female students cannot be effective if approached in isolation, through strictly nutritional measures; rather, it must obligatorily integrate psychological strategies aimed at reducing perceived stress, viewed as direct measures for preventing cardiometabolic risk.

### 4.1. Practical Recommendations for Clinicians and Educational Institutions

Based on the findings of this study, several actionable recommendations can be formulated for both clinical practice and academic environments:

- Implementation of Non-Invasive Screening Programs: It is highly recommended to utilize the WWI and the SAD-to-Waist Ratio in university student health centers as rapid, cost-effective, and substantially more precise instruments than raw BMI for the early identification of metabolic risks.
- Tailored Intervention Strategies Based on Academic Profiles: Because indices vary significantly among university programs (BS, CSE, and SSP), higher education institutions should design department-specific health campaigns. For instance, incorporating active breaks and optimizing ergonomics would benefit CSE students, who may be more predisposed to extreme physical inactivity.
- Integration of Psychological and Nutritional Support: Given the demonstrated impact of perceived stress (PSQ) on morphological indices, university nutrition or physical activity programs must not be implemented in isolation; instead, they should be obligatorily coupled with stress management techniques and psychological counseling.
- Monitoring Anthropometric Markers Throughout the University Years: It is strongly recommended to conduct longitudinal studies (tracking students from their first year until graduation) to precisely observe how exam-related stress and the academic lifestyle influence body shape evolution over time.
- Utilization of the Digit Ratio (2D:4D) in Complementary Research: Including this stable biological marker in screening evaluations can assist in identifying an underlying constitutional predisposition, thereby enabling a significantly more personalized and preventive healthcare approach.

### 4.2. Limitations of the Study

Although the present research provides valuable and original insights into the neuro-somatic interaction among young adults, several methodological limitations must be considered. First, the cross-sectional design of the study precludes the establishment of direct causal relationships between perceived stress and modifications in AVI. Second, utilizing a sample derived from a single higher education institution, limited to students from three specific academic profiles, restricts the generalizability of the findings to the broader population or other age cohorts. Third, the assessment of stress relied exclusively on psychological self-report scales, reflecting subjective perception, without integrating objective endocrine biomarkers (e.g., free cortisol). Finally, the absence of strict statistical control for variables related to detailed dietary behavior and precise physical activity levels represents a limitation that warrants adjustment in future research utilizing a longitudinal approach.

Furthermore, due to the comprehensive, sex- and program-stratified design of this study, a large number of statistical tests—including multiple correlation matrices and subgroup linear regressions—were performed. While family-wise error rates for mean comparisons were protected using the Tukey HSD adjustment, no formal alpha-level corrections (such as Bonferroni or False Discovery Rate) were applied to the regression sub-models. Consequently, the potential inflation of Type I error rates cannot be entirely ruled out. Readers are advised to interpret marginally significant *p*-values (e.g., those close to the 0.05 threshold) with caution, recognizing them as hypothesis-generating trends within specific demographic strata rather than definitive causal effects.

## 5. Conclusions

The present research highlights sex-dependent variations regarding the concurrent association of anthropometric parameters and psychological scores with elevated abdominal volume status within the analyzed cohort of university students:

- Baseline Anthropometric Parameter Alignment: Within both the male and female cohorts, standard anthropometric indices (BMI and BSA) maintain their status as primary baseline covariates, demonstrating high diagnostic accuracy within the screening models.
- Application of Advanced Waist Indices: The Weight-Adjusted Waist Index (WWI) represents a robust and cross-sex equilibrated instrument, suggesting that the monitoring of abdominal fat dynamics exhibits a balanced statistical pattern in both sexes through refined anthropometric equations in this demographic.
- Prenatal Digital and Regional Association Profiles: The fetal hormonal imprint (reflected by the R-2D:4D digit ratio) and regional geometric abdominal traceability (expressed by the SAD-to-Waist Ratio) are confirmed as statistically significant screening correlates specifically within the studied female student cohort, demonstrating limited or absent statistical association among the analyzed male student cohort.
- Psychosomatic Associations in Female Students: The study indicates the presence of a statistically significant concurrent relationship between the psychological and metabolic dimensions solely among the analyzed female students. The level of perceived stress acts as a valid concurrent correlate, showing that psychological indicators within this academic environment co-vary with the classification of visceral adiposity accumulation in the studied female student cohort, in contrast to male students.

## Figures and Tables

**Table 1:** Sample structure according to academic program and sex.

Academic Program	Men *n* (%)	Women *n* (%)	Total *n* (%)	*χ* ^2^	*p*
Biomedical Sciences	37 (25.7)	107 (74.3)	144 (100.0)	27.319	0.0001
Computer Science and Engineering	102 (64.2)	57 (35.8)	159 (100.0)	11.716	0.0006
Social Sciences and Physiotherapy	60 (42.3)	82 (57.7)	142 (100.0)	3.308	0.06
Total sample size	199 (44.7)	246 (55.3)	445 (100.0)	45.718	<0.001

Note: *n* = number of participants; % = percentage of the academic program total; *χ*^2^ = value of Pearson’s Chi-square test; *p* = statistical significance threshold.

**Table 2:** Descriptive and comparative profile (ANOVA) of anthropometric and psychological parameters based on sex and academic program.

Parameter	Sex	BS (*n* = 144) Mean ± SD	CSE (*n* = 159) Mean ± SD	SSP (*n* = 142) Mean ± SD	*F*	*p*	Post-Hoc (Tukey)
BMI	Men	27.78 ± 4.5	24.83 ± 4.61	24.75 ± 4.54	6.418	0.002	BS > CSE * BS > SSP *
	Women	24.66 ± 5.59	22.59 ± 4.72	23.44 ± 4.21	3.58	0.031	BS > CSE *
R-2D:4D	Men	0.98 ± 0.03	0.98 ± 0.03	0.97 ± 0.03	2.685	0.071	NS
	Women	1.00 ± 0.03	1.00 ± 0.21	1.00 ± 0.26	0.376	0.687	NS
SAD-to-Waist Ratio	Men	0.26 ± 0.02	0.26 ± 0.02	0.26 ± 0.02	0.225	0.779	NS
	Women	0.25 ± 0.02	0.26 ± 0.02	0.26 ± 0.02	4.332	0.014	BS < CSE *
BSA	Men	2.01 ± 0.17	1.95 ± 0.17	1.91 ± 0.17	4.115	0.018	BS > SSP *
	Women	1.70 ± 0.18	1.65 ± 0.17	1.71 ± 0.16	2.292	0.103	NS
WWI	Men	10.07 ± 0.60	9.85 ± 0.55	9.73 ± 0.65	3.696	0.027	BS > SSP *
	Women	9.50 ± 1.13	9.51 ± 0.76	9.41 ± 0.56	0.283	0.754	NS
PSQ	Men	61.24 ± 12.40	61.61 ± 12.31	63.53 ± 13.42	0.545	0.581	NS
	Women	67.22 ± 14.24	65.74 ± 13.05	66.99 ± 14.00	0.224	0.79	NS

Note: BS = Biomedical Sciences; CSE = Computer Science and Engineering; SSP = Social Sciences and Physiotherapy; BMI = Body Mass Index; R-2D:4D = Right Hand 2D:4D Digit Ratio; SAD-to-Waist Ratio = Sagittal Abdominal Diameter-to-Waist Circumference Ratio; BSA = Body Surface Area; WWI = Weight-Adjusted Waist Index; PSQ = Perceived Stress Questionnaire; NS = statistically non-significant. Means are rounded according to standard editorial requirements. * indicates significant differences at the *p* < 0.05 level in the post-hoc test.

**Table 3:** Pearson correlation matrix between anthropometric and psychological parameters, stratified by sex (Men above the diagonal/Women below the diagonal).

Coefficient	BMI	R-2D:4D	SAD-to-Waist Ratio	BSA	WWI	PSQ
BMI	1	0.238 **	0.374 **	0.736 **	0.639 **	−0.045
R-2D:4D	0.449 **	1	0.098	0.170 *	0.318 **	0.012
SAD-to-Waist Ratio	0.394 **	0.217 **	1	0.261 **	0.179 *	0.054
BSA	0.842 **	0.329 **	0.379 **	1	0.442 **	−0.021
WWI	0.454 **	0.286 **	0.130 *	0.326 **	1	0.062
PSQ	0.182 **	−0.012	0.041	0.038	0.029	1

Note: BMI = Body Mass Index; R-2D:4D = Right Hand 2D:4D Digit Ratio; SAD-to-Waist Ratio = Sagittal Abdominal Diameter-to-Waist Circumference Ratio; BSA = Body Surface Area; WWI = Weight-Adjusted Waist Index; PSQ = Perceived Stress Questionnaire. Values in the upper triangle (top-right) represent Pearson correlation coefficients (r) for men (*n* = 199). Values in the lower triangle (bottom-left) represent Pearson correlation coefficients (r) for women (*n* = 246). * Correlation is significant at the *p* < 0.05 level (2-tailed). ** Correlation is significant at the *p* < 0.01 level (2-tailed).

**Table 4:** Multilinear regression equation for concurrent association purposes based on academic program (criterion: AVI).

Sex	*R*	*R* ^2^	*F*	*df*	*p*
Male	0.993	0.986	2257.066	6	<0.001
Female	0.663	0.440	31.311	6	<0.001

Note: Criterion Variable: AVI; Covariates: BMI, R-2D:4D, SAD-to-Waist Ratio, BSA, WWI, PSQ.

**Table 5:** Global multiple linear regression models for assessing the Adiposity Volume Index (AVI) based on sex.

Sex	Covariates	Unstandardized Coefficients		Standardized Coefficients	*t*	*p*	Effect Size (f^2^)	Observed Power (1 − *β*)
		B	Std. Err.	β				
Male	Constant	−41.766	1.485	—	−28.118	<0.001	70.429	1.000 (100%)
	BMI	0.318	0.014	0.347	22.627	<0.001		
	R-2D:4D	0.032	0.083	0.003	0.384	0.701		
	SAD-to-Waist Ratio	5.335	2.090	0.024	2.553	0.011		
	BSA	9.663	0.311	0.393	31.110	<0.001		
	WWI	2.942	0.082	0.411	35.924	<0.001		
	PSQ	−0.010	0.008	−0.010	−1.189	0.236		
Female	Constant	1.363	12.169	—	0.112	0.911	0.786	1.000 (100%)
	BMI	0.401	0.114	0.362	3.520	0.001		
	R-2D:4D	−23.237	11.413	−0.112	−2.036	0.043		
	SAD-to-Waist Ratio	0.542	0.627	0.041	0.865	0.388		
	BSA	7.417	2.975	0.228	2.493	0.013		
	WWI	1.574	0.345	0.251	4.562	<0.001		
	PSQ	0.012	0.014	0.040	0.857	0.395		

Note: BMI = Body Mass Index; R-2D:4D = Right Hand 2D:4D Ratio; SAD-to-Waist Ratio = Sagittal Abdominal Diameter to Waist Circumference Ratio; BSA = Body Surface Area; WWI = Weight-Adjusted Waist Index; PSQ = Perceived Stress Questionnaire.

**Table 6:** Multiple linear regression models for assessing the AVI, stratified by sex and academic program.

Sex	Academic Program	Covariates	Unstandardized Coefficients		Standardized Coefficients	*t*	*p*	Effect Size (f^2^)	Observed Power (1 − *β*)
			*B*	Std. Err.	*β*				
Male	BS *R*^2^ = 0.989, *p* < 0.001	Constant	−46.909	3.340	-	−14.043	<0.001	89.909	1.000 (100%)
		BMI	0.253	0.030	0.277	8.508	<0.001		
		SAD-to-Waist Ratio	11.067	4.988	0.051	2.219	0.034		
		BSA	11.844	0.674	0.475	17.575	<0.001		
		WWI	3.056	0.164	0.449	18.672	<0.001		
		PSQ	−0.020	0.007	−0.059	−2.901	0.007		
	CSE *R*^2^ = 0.986, *p* < 0.001	Constant	−42.525	2.365	-	−17.982	<0.001	70.429	1.000 (100%)
		BMI	0.323	0.021	0.360	15.369	<0.001		
		BSA	9.702	0.485	0.408	19.985	<0.001		
		WWI	2.943	0.120	0.391	24.437	<0.001		
	SSP *R*^2^ = 0.988, *p* < 0.001	Constant	−40.410	2.475	-	−16.328	<0.001	82.333	1.000 (100%)
		BMI	0.332	0.027	0.345	12.371	<0.001		
		SAD-to-Waist Ratio	9.969	3.293	0.051	3.027	0.004		
		BSA	8.925	0.532	0.353	16.769	<0.001		
		WWI	2.877	0.149	0.430	19.344	<0.001		
Female	BS *R*^2^ = 0.296, *p* < 0.001	Constant	26.719	26.962	-	0.991	0.324	0.420	1.000 (100%)
		WWI	1.420	0.617	0.218	2.303	0.023		
		Note: Both BMI (*p* = 0.093) and R-2D:4D (*p* = 0.056) did not reach the significance threshold							
	CSE *R*^2^ = 0.976, *p* < 0.001	Constant	−40.493	4.819	-	−8.403	<0.001	40.667	1.000 (100%)
		BMI	0.205	0.041	0.246	4.980	<0.001		
		BSA	10.372	0.970	0.461	10.695	<0.001		
		WWI	2.699	0.143	0.522	18.903	<0.001		
	SSP *R*^2^ = 0.991, *p* < 0.001	Constant	−27.794	1.879	-	−14.789	<0.001	110.111	1.000 (100%)
		BMI	0.313	0.021	0.406	14.624	<0.001		
		BSA	7.886	0.441	0.388	17.868	<0.001		
		WWI	2.240	0.082	0.384	27.449	<0.001		

Note: BS = Biomedical Sciences; CSE = Computer Science and Engineering; SSP = Social Sciences and Physiotherapy; BMI = Body Mass Index; R-2D:4D = Right Hand 2D:4D Digit Ratio; SAD-to-Waist Ratio = Sagittal Abdominal Diameter-to-Waist Circumference Ratio; BSA = Body Surface Area; WWI = Weight-Adjusted Waist Index; PSQ = Perceived Stress Questionnaire. For conciseness, only covariates that reached the statistical significance threshold within each sub-model (*p* < 0.05) or displayed major trends are presented.

**Table 7:** Multiple linear regression models for assessing the AVI, stratified by sex and the presence of psychological stress.

Sex	Stress Status	Covariates	Unstandardized Coefficients		Standardized Coefficients	*t*	*p*	Effect Size (f^2^)	Observed Power (1 − *β*)
			B	Std. Err.	β				
Male	LLS (0.00 score) (*R*^2^ = 0.987, *p* < 0.001)	BMI	0.343	0.018	0.378	19.043	<0.001	75.923	1.000 (100%)
		BSA	9.574	0.404	0.390	23.701	<0.001		
		WWI	2.716	0.118	0.356	23.003	<0.001		
	HLS (1.00 score) (*R*^2^ = 0.986, *p* < 0.001)	BMI	0.284	0.023	0.305	12.596	<0.001	70.429	1.000 (100%)
		BSA	9.951	0.484	0.403	20.581	<0.001		
		WWI	3.156	0.114	0.482	27.760	<0.001		
Female	LLS (0.00 score) (*R*^2^ = 0.883, *p* < 0.001)	BMI	0.484	0.048	0.645	9.990	<0.001	7.547	1.000 (100%)
		BSA	5.046	1.185	0.249	4.258	<0.001		
		WWI	0.564	0.115	0.187	4.898	<0.001		
	HLS (1.00 score) *R*^2^ = 0.976, *p* < 0.001	SAD-to-Waist Ratio	−45.463	18.547	−0.186	−2.451	0.015	40.667	1.000 (100%)
		BSA	10.906	5.206	0.290	2.095	0.038		
		WWI	3.871	0.770	0.396	5.029	<0.001		

Note: LLS (0.00 score) = Low Level of Stress; HLS (1.00 score) = High Level of Stress; BMI = Body Mass Index; SAD-to-Waist Ratio = Sagittal Abdominal Diameter-to-Waist Circumference Ratio; BSA = Body Surface Area; WWI = Weight-Adjusted Waist Index. The criterion (dependent variable) is AVI. For conciseness, only covariates that reached the statistical significance threshold within each stratified subgroup (*p* < 0.05) are presented.

**Table 8:** Multiple linear regression models for assessing the AVI, stratified three-dimensionally by sex, academic program, and stress status.

Sex	Academic Program	Stress Status	Covariates	Unstandardized Coefficients		Standardized Coefficients	*t*	*p*	Effect Size (f^2^)	Observed Power (1 − *β*)
				*B*	Std. Err.	*β*				
Male	BS	LLS (0.00 score) *R*^2^ = 0.989	BMI	0.224	0.039	0.256	5.697	<0.001	89.909	1.000 (100%)
			SAD-to-Waist Ratio	15.303	7.146	0.060	2.141	0.047		
			BSA	13.441	1.026	0.490	13.102	<0.001		
			WWI	2.908	0.232	0.394	12.553	<0.001		
		HLS (1.00 score) *R*^2^ = 0.986	BMI	0.291	0.042	0.273	6.894	<0.001	89.909	1.000 (100%)
			BSA	9.462	0.708	0.460	13.369	<0.001		
			WWI	3.283	0.185	0.570	17.741	<0.001		
	CSE	LLS (0.00 score) *R*^2^ = 0.986	BMI	0.359	0.024	0.388	14.995	<0.001	70.429	70.429
			BSA	9.513	0.549	0.404	17.339	<0.001		
			WWI	2.739	0.153	0.332	17.931	<0.001		
		HLS (1.00 score) *R*^2^ = 0.978	BMI	0.283	0.037	0.329	7.621	<0.001	44.455	1.000 (100%)
			BSA	10.012	0.889	0.414	11.262	<0.001		
			WWI	3.113	0.193	0.465	16.106	<0.001		
	SSP	LLS (0.00 score) *R*^2^ = 0.988	BMI	0.352	0.035	0.394	10.046	<0.001	82.333	1.000 (100%)
			BSA	8.450	0.686	0.353	12.310	<0.001		
			WWI	2.648	0.233	0.383	11.372	<0.001		
		HLS (1.00 score) *R*^2^ = 0.988	BMI	0.326	0.045	0.305	7.238	<0.001	82.333	1.000 (100%)
			BSA	10.046	0.889	0.470	11.296	<0.001		
			WWI	3.083	0.186	0.470	16.553	<0.001		
Female	BS	LLS (0.00 score) *R*^2^ = 0.908	BMI	0.299	0.086	0.400	3.485	0.001	9.870	1.000 (100%)
			R-2D:4D	25.603	8.970	0.184	2.854	0.007		
			BSA	7.959	2.141	0.360	3.718	0.001		
		HLS (1.00 score) *R*^2^ = 0.313	R-2D:4D	−87.780	36.264	−0.304	−2.421	0.019	0.456	0.952 (95.2%)
			WWI	5.378	1.636	0.428	3.288	0.002		
	CSE	LLS (0.00 score) *R*^2^ = 0.988	BMI	0.255	0.042	0.368	6.093	<0.001	82.333	1.000 (100%)
			BSA	8.402	0.981	0.457	8.567	<0.001		
			WWI	2.104	162	0.392	12.969	<0.001		
		HLS (1.00 score) *R*^2^ = 0.978	BMI	0.157	0.059	0.169	2.657	0.013	44.455	1.000 (100%)
			BSA	12.177	1.390	0.481	8.762	<0.001		
			WWI	2.862	0.198	0.544	14.464	<0.001		
	SSP	LLS (0.00 score) *R*^2^ = 0.989	BMI	0.345	0.035	0.399	9.974	<0.001	89.909	1.000 (100%)
			BSA	6.865	0.552	0.374	12.432	<0.001		
			WWI	2.068	0.123	0.518	16.824	<0.001		
		HLS (1.00 score) *R*^2^ = 0.993	BMI	0.282	0.031	0.370	8.988	<0.001	141.857	1.000 (100%)
			BSA	8.802	0.736	0.424	11.957	<0.001		
			WWI	2.150	0.080	0.380	26.875	<0.001		

Note: BS = Biomedical Sciences; CSE = Computer Science and Engineering; SSP = Social Sciences and Physiotherapy; BMI = Body Mass Index; R-2D:4D = Right Hand 2D:4D Digit Ratio; SAD-to-Waist Ratio = Sagittal Abdominal Diameter-to-Waist Circumference Ratio; BSA = Body Surface Area; WWI = Weight-Adjusted Waist Index; LLS (0.00 score) = Low Level of Stress; HLS (1.00 score) = High Level of Stress. The model is simultaneously stratified across three levels. To maintain clarity, only predictors with statistical significance within each subgroup (*p* < 0.05) are presented. All global models were highly significant at the *p* < 0.001 threshold.

**Table 9:** Discriminatory capacity and optimal cut-off thresholds of anthropometric and psychological covariates in identifying elevated adiposity volume (AVI threshold based on clinical criteria).

Sex	Covariates	*AUC*	95% CI	*p*	Cut-Off	Sensitivity	Specificity	Youden Index
Male	BMI	0.957	0.928–0.987	<0.001	26.96	87.3%	91.9%	0.681
	WWI	0.941	0.909–0.972	<0.001	10.12	87.3%	89.0%	0.735
	BSA	0.911	0.870–0.951	<0.001	1.9974	84.1%	86.0%	0.589
	R-2D:4D	0.680	0.598–0.762	<0.001	0.9945	55.6%	77.9%	0.143
	SAD-to-Waist Ratio	0.633	0.549–0.717	<0.003	0.2568	61.9%	60.3%	0.089
	PSQ	0.545	0.462–0.628	0.306	57.50	61.9%	44.1%	−0.042
Female	BMI	0.972	0.953–0.991	0.001	24.89	90.9%	91.7%	0.826
	BSA	0.946	0.918–0.973	<0.001	1.74	89.4%	90.0%	0.794
	WWI	0.910	0.873–0.947	<0.001	9.75	83.3%	86.7%	0.700
	R-2D:4D	0.797	0.731–0.864	<0.001	1.0077	80.3%	75.0%	0.553
	SAD-to-Waist Ratio	0.680	0.603–0.758	<0.001	0.2528	71.2%	51.51	0.223
	PSQ	0.621	0.543–0.698	0.004	65.50	63.6%	53.9%	0.175

Note: BMI = Body Mass Index; R-2D:4D = Right Hand 2D:4D Digit Ratio; SAD-to-Waist Ratio = Sagittal Abdominal Diameter-to-Waist Circumference Ratio; BSA = Body Surface Area; WWI = Weight-Adjusted Waist Index; PSQ = Perceived Stress Questionnaire. The binary criterion utilized is represented by the AVI variable binarized dichotomized using externally validated clinical risk thresholds.

## Data Availability

The data presented in this study are available on request from the corresponding author. The data are not publicly available due to privacy restrictions and ethical restrictions.

## Abbreviations

BMI	Body Mass Index
BSA	Body Surface Area
2D	2nd digit (index finger)
4D	4th digit (ring finger)
R-2D:4D	Right Hand 2D:4D Ratio
SAD-to-Waist Ratio	Sagittal Abdominal Diameter to Waist Circumference Ratio
WWI	Weight-Adjusted Waist Index
AVI	Abdominal Volume Index
SAD	Sagittal Abdominal Diameter
W	Weight
H	Height
WC	Waist Circumference
HC	Hip Circumference
NWO	Normal-Weight Obesity
PSQ	Perceived Stress Questionnaire
LLS (0.00 score)	Low Level of Stress
HLS (1.00 score)	High Level of Stress
BS	Biomedical Sciences
CSE	Computer Science and Engineering
SSP	Social Sciences and Physiotherapy
ANOVA	Analysis of Variance
ROC	Receiver Operating Characteristic
*AUC*	Area Under the Curve
CI	Confidence Interval
SD	Standard Deviation
HPA	Hypothalamic–Pituitary–Adrenal Axis
